# Albumin Level at Admission to the Intensive Care Unit Is Associated With Prognosis in Cardiac Arrest Patients

**DOI:** 10.7759/cureus.14501

**Published:** 2021-04-15

**Authors:** Yide Li, Yingfang She, Weisheng Mo, Biao Jin, Wendi Xiang, Liang Luo

**Affiliations:** 1 Department of Critical Care Medicine, The Seventh Affiliated Hospital of Sun Yat-sen University, Shenzhen, CHN; 2 Neurology Medicine Center, The Seventh Affiliated Hospital of Sun Yat-sen University, Shenzhen, CHN; 3 Department of Operating Room, Xiangya Hospital of Central South University, Changsha, CHN

**Keywords:** albumin, cardiac arrest, eicu-crd

## Abstract

Aim

Cardiac arrest is a global health concern with consistently high mortality. It is also a common condition seen in the intensive care unit (ICU). We aimed to investigate the importance of albumin level on admission, which is a widely available and simple test, to predict in-hospital mortality in cardiac arrest patients.

Methods

The retrospective study collected data from the eICU Collaborative Research Database. It contains data from 171 hospitals, 276 ICU wards, and 4,529 patients who were treated for cardiac arrest from 2014 to 2015. We analyzed the patients’ laboratory results and vital signs during the first 24 hours after admission to the ICU. The primary outcome was in-hospital mortality, and the secondary outcome was the length of ICU stay among survivors.

Results

In total, 2,414 patients were eligible. After adjusting for severity scores, including the Acute Physiology and Chronic Health Evaluation (APACHE) IV and Sequential Organ Failure Assessment (SOFA) scores, serum albumin was found to be a protective factor for survival (odds ratio of mortality: 0635, 95% confidence interval: 0.458-0.734, P<0.001). Among patients who survived until discharge, those with hypoalbuminemia had a long duration of stay in the ICU (P=0.005).

Conclusion

The higher albumin level at admission to the ICU was associated with lower mortality in patients with cardiac arrest.

## Introduction

Cardiac arrest is the sudden interruption of cardiac blood flow in a person who may or may not have been diagnosed with a cardiac disease before. It is a global health concern that has a consistently high mortality. It was estimated that among 347,322 cases of out-of-hospital cardiac arrest (OHCA) in the U.S., the survival rate until hospital admission after emergency medical service-treated non-traumatic OHCA was 29%. Among the adult patients, the survival rate until hospital discharge was 10.8% [1]. In contrast, the survival rate of in-hospital cardiac arrest (IHCA) patients was about 20% [2]. Although the 1-year survival rate of OHCA patients after discharge is increasing in recent times (8.0% in 2000-2009 vs. 13.3% in 2010-2019) [3], it is still far from ideal. Due to the high in-hospital mortality, distinguishing the high-risk patients at admission by some practical tools is important. Some scores were developed to predict the outcomes among OHCA patients in the hospital. However, these methods were not efficient [4] or based on small sample sizes [5].

Serum albumin is a part of the hepatic function test and is routinely assessed at admission in critical patients. Albumin level is a strong predictor of many diseases and surgeries [6], particularly in some critical illnesses, such as sepsis [7] and acute myocardial infarction [8], and also in critically ill children [9]. Albumin level might also be a predictor of prognosis in cardiac arrest patients. However, a study with a large sample is necessary to confirm this hypothesis. This study aimed to investigate the importance of albumin level, which is a widely available and simple test, to predict in-hospital mortality in cardiac arrest patients.

## Materials and methods

Study population

The retrospective study involved 171 hospitals, 276 intensive care unit (ICU) wards, and 4,529 patients. All the patients’ data were collected from the eICU Collaborative Research Database (eICU-CRD) [10]. It is a large, multicentre ICU database that contains data for over 200,000 admissions to ICUs observed by the eICU programs across the United States in 2014 and 2015. The eICU Program was a remote health system developed by Philips Healthcare. The Laboratory for Computational Physiology (LCP) at the Massachusetts Institute of Technology (MIT) participated in establishing the eICU-CRD. The database was released with the patients’ identities completely masked, under the Health Insurance Portability and Accountability Act safe harbor provision. Since the study was a retrospective investigation of a third-party, anonymized, publicly available database with pre-existing institutional review board (IRB) approval, the requirement for IRB approval from our institution was exempted.

Criteria for selecting the subjects were as follows:

i. The primary diagnosis was cardiac arrest at admission to the ICU;

ii. Age >16 years and age <89 years;

iii. First admission to ICU;

iv. Duration of ICU stay ≥4 hours;

v. Integrity of key information (gender, Acute Physiology and Chronic Health Evaluation [APACHE] IV scores, mortality, and albumin level within the first 24 hours in the ICU)

Data, including demographic characteristics, mortality, disease severity scores, Charlson comorbidity index, laboratory results, length of ICU stay, and vital signs, were collected from the eICU-CRD. If there were more than one laboratory results recorded for the same patient within the first 24 hours of ICU admission, only the first result was included in the study. Additionally, the vital signs recorded at ICU admission were included. The disease severity scores included the Sequential Organ Failure Assessment (SOFA) and APACHE IV scores. The SOFA source code was shared by Johnson et al. [11].

Statistical analysis

Continuous variables were tested for normality by the Shapiro-Wilk test. None of them conformed to the normal distribution. Data were presented as either frequency and percentage for categorical variables or median and interquartile range (IQR) for continuous variables. The Kruskal-Wallis H test was used to compare continuous data. Categorical variables were compared with Fisher’s exact test. The receiver operating characteristic (ROC) curve and multivariable logistic regression models were used to investigate the possible association between albumin and hospital mortality. Hours of ICU stay in survival patients were compared between patients with albumin <3.5 g/dL and ≥3.5 g/dL by using the Log-rank test. All significance tests were performed as two-sided, and a P-value of <0.05 was considered statistically significant. All analyses were conducted using R 3.6.2 (R Foundation, Vienna, Austria) with the pROC [12] and ggplot2 packages.

## Results

**Figure 1:**
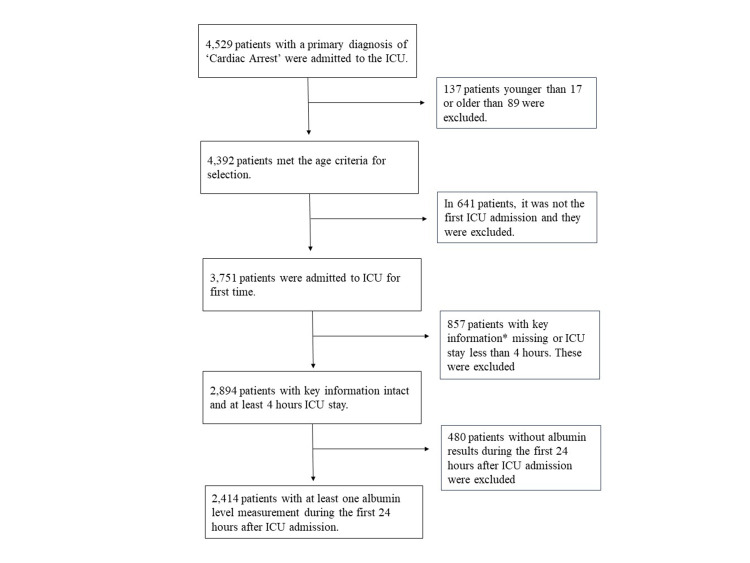
Patient selection * The key information refers to gender, mortality, APACHE IV scores. ICU: intensive care unit.

**Figure 2:**
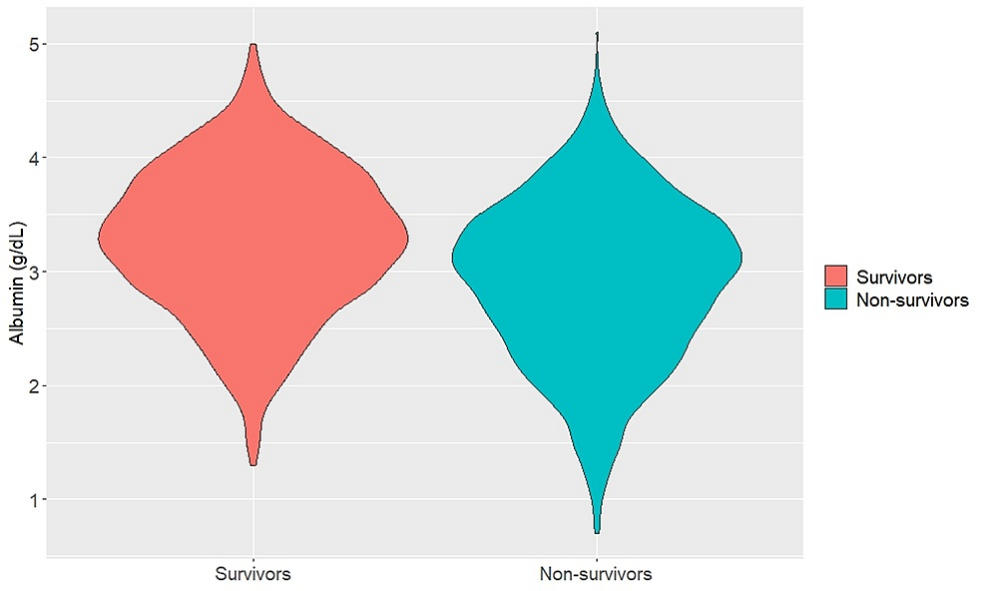
Comparison of albumin between survivors and non-survivors

In total, 2,414 patients were eligible (Figure 1) for the analysis. Their demographic and clinical characteristics are summarized in Table 1. There were 1,065 survivors and 1,349 non-survivors during the hospital stay. The non-survivors had higher disease severity scores and Charlson comorbidity indexes than the survivors. Among the laboratory tests, non-survivors had a higher deviation from the normal values for parameters such as red cell distribution width, albumin, creatinine, and lactate. A comparison of albumin levels between the two groups of patients is shown in Figure 2.

**Table 1:** Baseline characteristics of total patients, survivors, and non-survivors *Due to the missing data, the sum of the patients was not equal to the actual number. ICU: intensive care unit; RDW: red blood distribution width; MCH: mean corpuscular hemoglobin; MCHC: Mean corpuscular hemoglobin concentration; BUN: blood urea nitrogen; WBC: white blood cells; BMI: body mass index; MAP: mean arterial pressure.

	All	Survivors	Non-Survivors	p
Numbers	2414	1065	1349	
Age (year)	64.00 [53.00, 74.00]	63.00 [52.00, 73.00]	66.00 [55.00, 75.00]	<0.001
Female (%)	1035 (42.9)	437 (41.0)	598 (44.3)	0.113
Ethnicity* (%)				0.126
African American	358 (15.1)	174 (16.6)	184 (13.9)	
Asian	42 ( 1.8)	20 ( 1.9)	22 ( 1.7)	
Caucasian	1752 (73.8)	755 (71.9)	997 (75.2)	
Hispanic	87 ( 3.7)	36 ( 3.4)	51 ( 3.8)	
Native American	21 ( 0.9)	6 ( 0.6)	15 ( 1.1)	
Other/Unknown	115 ( 4.8)	59 ( 5.6)	56 ( 4.2)	
Length of ICU stay	66.00 [28.00, 128.00]	90.00 [47.00, 170.00]	49.00 [18.00, 99.00]	<0.001
SOFA Scores	8.00 [5.00, 11.00]	6.00 [4.00, 9.00]	9.00 [7.00, 12.00]	<0.001
Charlson comorbidity index	4.00 [2.00, 5.00]	3.00 [1.00, 5.00]	4.00 [2.00, 6.00]	<0.001
Apache Ⅳ Scores	107.00 [78.00, 130.75]	84.00 [56.00, 111.00]	121.00 [99.00, 141.00]	<0.001
RDW (%)	14.70 [13.50, 16.50]	14.30 [13.40, 16.00]	15.00 [13.80, 16.88]	<0.001
MCH (pg),	30.00 [28.30, 31.33]	30.00 [28.40, 31.40]	30.00 [28.30, 31.30]	0.231
MCHC (g/dl)	32.30 [31.10, 33.20]	32.70 [31.60, 33.70]	32.00 [30.90, 33.00]	<0.001
Albumin (g/dl)	3.10 [2.60, 3.50]	3.30 [2.90, 3.70]	3.00 [2.40, 3.40]	<0.001
Bicarbonate (mmol/L)	21.00 [18.00, 25.00]	22.60 [19.00, 26.00]	20.00 [17.00, 25.00]	<0.001
Total bilirubin (mg/dl)	0.60 [0.40, 0.90]	0.50 [0.40, 0.80]	0.60 [0.40, 0.90]	0.12
Creatinine (mg/dl)	1.40 [1.00, 2.12]	1.25 [0.91, 1.86]	1.50 [1.10, 2.30]	<0.001
Chloride (mmol/L)	102.00 [98.00, 106.00]	102.00 [99.00, 106.00]	102.00 [97.00, 106.00]	0.02
Glucose (mg/dl)	192.00 [131.00, 276.00]	173.00 [124.00, 243.50]	211.00 [138.50, 294.50]	<0.001
Hematocrit (%),	37.50 [32.20, 42.50]	38.00 [33.10, 42.70]	36.90 [31.40, 42.23]	0.001
Hemoglobin (g/dl)	12.10 [10.20, 13.90]	12.50 [10.50, 14.10]	11.70 [9.90, 13.60]	<0.001
Lactate (mmol/L)	5.10 [2.40, 9.20]	3.40 [1.70, 5.90]	6.70 [3.50, 10.90]	<0.001
Platelet (*10^9^/L)	213.00 [164.00, 275.00]	222.00 [176.00, 279.00]	208.00 [152.00, 268.00]	<0.001
Potassium (mmol/L)	4.20 [3.70, 4.90]	4.10 [3.60, 4.70]	4.30 [3.80, 5.10]	<0.001
Sodium (mmol/L)	138.00 [135.00, 141.00]	138.00 [135.00, 141.00]	139.00 [135.00, 142.00]	0.152
BUN (mmol/L)	22.00 [15.00, 36.00]	20.00 [14.00, 32.00]	23.00 [16.00, 38.00]	<0.001
WBC (*10^9^/L)	12.48 [8.90, 17.40]	12.10 [8.80, 16.40]	12.90 [9.00, 18.10]	0.005
Heart Rate	90.00 [74.00, 108.00]	88.00 [73.00, 104.00]	92.00 [75.00, 109.00]	<0.001
Respiratory Rate	20.00 [16.00, 24.00]	19.00 [16.00, 23.00]	20.00 [16.00, 25.00]	<0.001
Temperature (℃)	36.20 [34.70, 36.80]	36.40 [35.70, 36.90]	35.70 [34.30, 36.60]	<0.001
BMI (kg/m^2^)	28.51 [24.33, 34.17]	28.69 [24.75, 33.84]	28.45 [24.01, 34.29]	0.44
MAP (mmHg)	84.30 [70.70, 100.00]	87.70 [74.70, 101.60]	81.00 [67.70, 98.70]	<0.001

**Figure 3:**
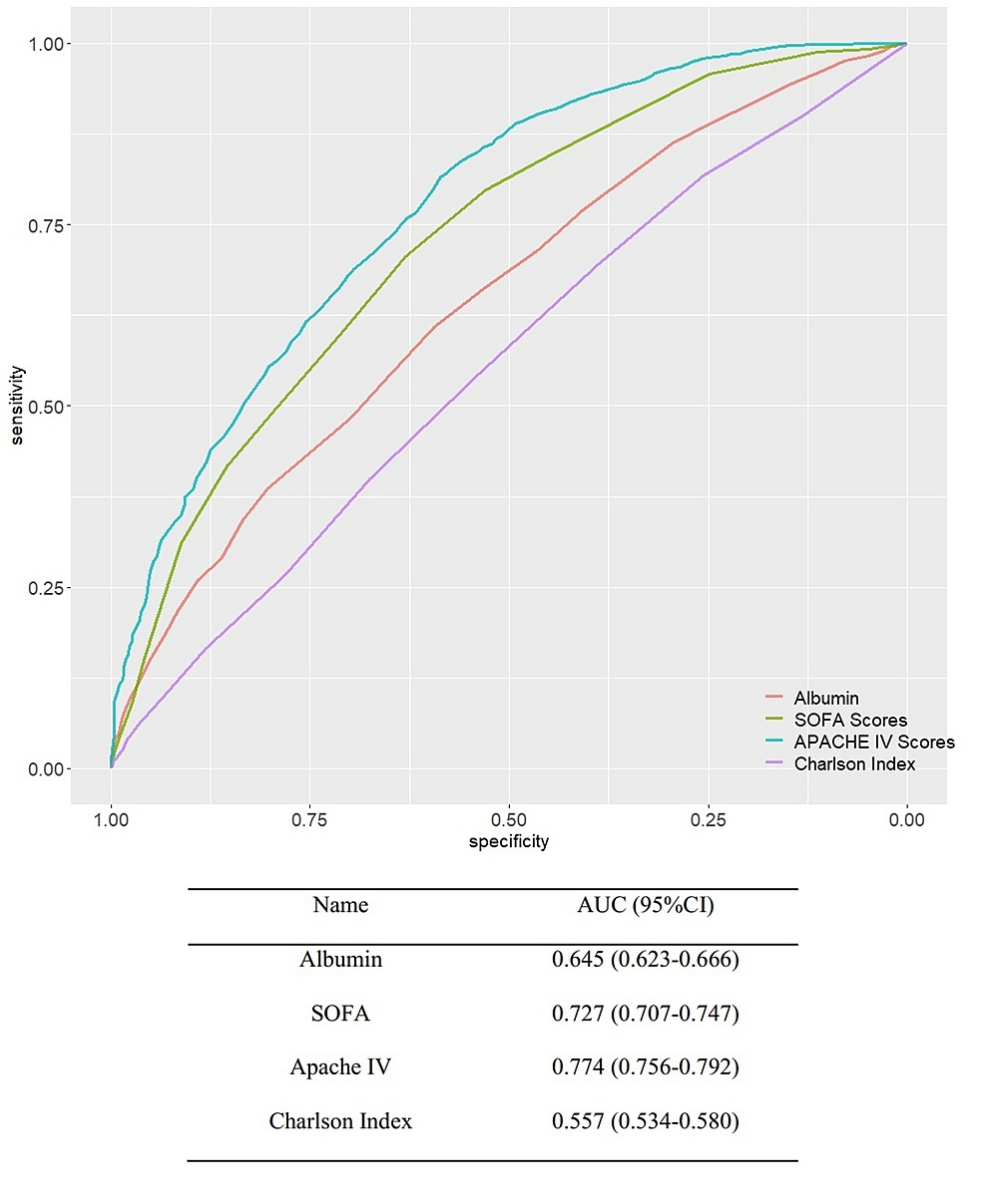
ROCs of albumin and disease severity scores for hospital mortality AUC: area under the curve; ROC: receiver operating characteristic.

Receiver operating characteristic analysis was performed to examine the correlation between albumin level and in-hospital mortality. The ROC curves for red cell distribution width and severity-of-disease scores are depicted in Figure 3, and the areas under the curve (AUCs) are presented in Table 2. The reliability of albumin levels in predicting the in-hospital mortality was poorer than that of the SOFA and APACHE IV scores, which are considered as classic severity scores; however, the reliability of albumin levels was better than that of the Charlson comorbidity index.

**Table 2:** AUCs of ROC for albumin and disease severity scores (Delong test) AUC: area under the curve; ROC: receiver operating characteristic

Name	AUC (95%CI)
Albumin	0.645 (0.623-0.666)
SOFA score	0.727 (0.707-0.747)
Apache IV score	0.774 (0.756-0.792)
Charlson comorbidity index	0.557 (0.534-0.580)

**Figure 4:**
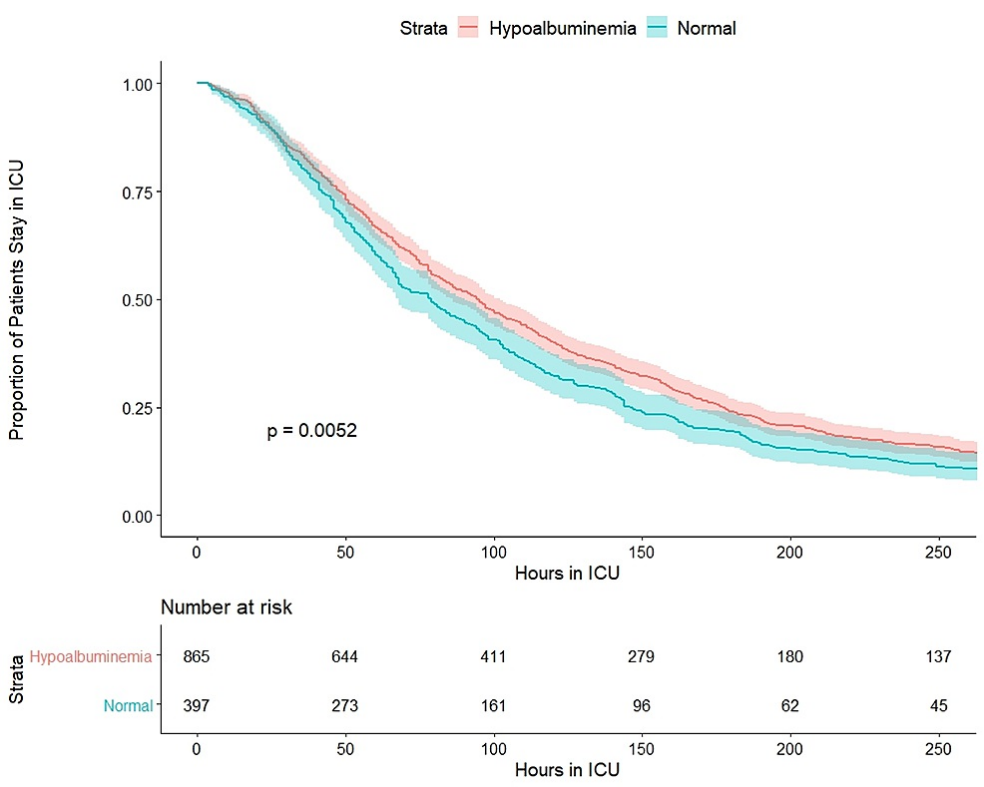
Kaplan-Meier survival curves for comparison of ICU length of stay between survivors with hypoalbuminemia and normal albumin levels ICU: intensive care unit

We performed multiple logistic regression analyses to investigate the association between albumin and in-hospital mortality in the study population. After adjusting for the SOFA scores, APACHE IV scores, and Charlson comorbidity index, a significant negative correlation was found between the albumin level and in-hospital mortality (Table 3). Figure 4 shows the Kaplan-Meier survival estimates for ICU length of stay in patients with hypoalbuminemia and those with normal albumin levels among the survivors. The result revealed that hypoalbuminemia was associated with a significantly longer length of stay in the ICU (Log-rank test, P=0.005).

**Table 3:** Multivariable logistic regression analysis for in-hospital mortality OR: odds ratio; CI: confidence interval.

	OR (95%CI)	P
Albumin (per 1 g/dl)	0.635 (0.458-0.734)	<0.001
SOFA score (per 1 score)	1.039 (1.003- 1.075)	0.032
Apache IV score (per 1 score)	1.028 (1.024-1.033)	<0.001
Charlson comorbidity index (per 1 score)	1.015 (0.980-1.052)	0.397

## Discussion

This study focused on the relationship between albumin levels at admission in the ICU and prognosis in patients with cardiac arrest. Our study showed that lower albumin level at admission was associated with increased hospital mortality and prolonged length of ICU stay. Although the AUC of the ROC for albumin was lower than that of the APACHE IV and SOFA scores, it had a significant predictive performance.

In this prospective study, we involved multiple hospitals throughout the United States. The in-hospital mortality of the study population was 55.88%, which was lower than that mentioned in the previous literature [1]. The reason for this might be that only relatively stable patients can be transferred from the emergency department to the ICU in most cases. However, their mortality rate was relatively higher compared to other critically ill patients in the ICU. Hence, early identification of high-risk patients is necessary. Since hypoalbuminemia might be the result of a pre-existing chronic disease and comorbidities before the cardiac arrest, we adjusted the APACHE IV score, SOFA score, and Charlson Comorbidity index as confounders. Albumin was still found to be an independent protective factor for prognosis.

Albumin is the principal plasma protein and has multiple physiological functions [13]. It interacts with water, cations, fatty acids, hormones, bilirubin, thyroxine, and pharmaceuticals. Its main function is to maintain the osmotic pressure of blood. Hypoalbuminemia can occur due to different causes such as decreased production, increased loss, increased use in the body, or abnormal distribution of albumin between the body parts [14]. In cardiac arrest, vascular permeability for the cells and plasma solutes is increased as a common reaction, and albumin might leak out of the vessels. Moreover, albumin kinetics get altered in the process [15]. Furthermore, due to the systemic inflammatory cascade and systemic ischemia-reperfusion response after cardiac arrest, many endotoxins and free radicals are produced. Albumin acts as a scavenger of reactive oxygen radicals and reactive nitrogen species through its ability to bind and transport [16]. Consequently, hypoalbuminemia might deteriorate further within several hours after resuscitation in previously healthy individuals. Hypoalbuminemia is associated with decreased muscle mass, organ function, and cognitive and immune function [17, 18], which can lead to a poor prognosis. It has been proved that hypoalbuminemia is associated with poor outcomes in many conditions like acute coronary syndrome [19], surgery [20-22], stroke [23, 24], and cancer [25]. Few previous studies have investigated the relationship between albumin levels and prognosis in cardiac arrest patients. Matsuyama found that hypoalbuminemia is associated with worse neurologic outcomes in patients with OHCA [26], which was based on 1,269 patients in Japan. However, this study did not consider the influence of previous chronic diseases, while our study considered APACHE IV score as a confounder, which provides chronic health evaluation [27].

The study has some limitations. First, we did not distinguish between OHCA and IHCA. However, one of the characteristics of this study is that the research population consisted of scenarios that ICU doctors encounter in the real world. Second, we did not assess whether infusing albumin products could reduce mortality. It might be a promising subject but is controversial [28]. Third, we do not have follow-up data after discharge from the hospital. The long-term outcome of patients who survive cardiac arrest is equally important. Fourth, this study may have spared the reaction of other latent confounders.

## Conclusions

The higher albumin level at admission to ICU is associated with lower mortality in cardiac arrest patients. The serum albumin is a protective factor to hospital mortality (OR:0635, 95%CI: 0.458-0.734, P<0.001). Among patients alive at discharge, hypoalbuminemia patients had a longer stay time in ICU (P=0.0052). We suggest that albumin level might be useful to predict mortality in patients with cardiac arrest.
